# Lysyl oxidase family activity promotes resistance of pancreatic ductal adenocarcinoma to chemotherapy by limiting the intratumoral anticancer drug distribution

**DOI:** 10.18632/oncotarget.8527

**Published:** 2016-04-01

**Authors:** Benjamin Le Calvé, Audrey Griveau, David Vindrieux, Raphaël Maréchal, Clotilde Wiel, Magali Svrcek, Johann Gout, Lamia Azzi, Léa Payen, Jérôme Cros, Christelle de la Fouchardière, Pierre Dubus, Jérôme Guitton, Laurent Bartholin, Jean-Baptiste Bachet, David Bernard

**Affiliations:** ^1^ Inserm U1052, Centre de Recherche en Cancérologie de Lyon, Lyon, France; ^2^ CNRS UMR5286, Lyon, France; ^3^ Centre Léon Bérard, Lyon, France; ^4^ Université de Lyon, Lyon, France; ^5^ Department of Gastroenterology and Gastrointestinal Cancer Unit, Erasme Hospital, Université Libre de Bruxelles, Belgium; ^6^ Department of Pathology, AP-HP, Hôpitaux Universitaires Est Parisien, Saint-Antoine Hospital, Paris, France; ^7^ Sorbonne University, UPMC University, Paris, France; ^8^ Service de Biologie des Tumeurs, CHU de Bordeaux Hôpital du Haut Lévêque, Pessac, France; ^9^ Hospices Civils de Lyon, Université de Lyon, Lyon, France; ^10^ AP-HP, Hôpitaux Universitaires Paris Nord Val de Seine, Beaujon, France; ^11^ Paris Diderot University, Paris, France; ^12^ Sorbonne University, UPMC University and INSERM, UMRS 1147, Paris Descrates University, Paris, France; ^13^ Gastroenterology Department, APHP, Pitié Salpêtrière Hospital, Paris, France; ^14^ Present address: URBC-NARILIS, University of Namur, Namur, Belgium

**Keywords:** chemoresistance, collagen, survival, biomarker, lysyl oxidase

## Abstract

Solid tumors often display chemotherapy resistance. Pancreatic ductal adenocarcinoma (PDAC) is the archetype of resistant tumors as current chemotherapies are inefficient. The tumor stroma and extracellular matrix (ECM) are key contributors to PDAC aggressiveness and to limiting the efficacy of chemotherapy. Lysyl oxidase (LOX) family members mediate collagen cross-linking and thus promote ECM stiffening. Our data demonstrate increased LOX, LOXL1, and LOXL2 expression in PDAC, and that the level of fibrillar collagen, which is directly dependent of LOX family activity, is an independent predictive biomarker of adjuvant “Gemcitabine-based chemotherapy” benefit. Experimentally in mice, increased LOX family activity through LOXL2 promotes chemoresistance. This effect of LOX family activity seems to be due to decreased gemcitabine intra-tumoral diffusion. This observation might be explained by increased fibrillar collagen and decreased vessel size observed in tumors with increased LOX family activity. In conclusion, our data support that LOX family activity is both a novel target to improve chemotherapy as well as a novel biomarker to predict gemcitabine benefit in PDAC. Beyond the PDAC, it is possible that targeting LOX family activity might improve efficacy of chemotherapies against different kinds of solid tumors.

## INTRODUCTION

Pancreatic ductal adenocarcinoma (PDAC) is one of the worst cancers, with a low five-year survival rate. It is currently the fifth cause of cancer mortality in the USA and its incidence could increase, to make it one of the top-ranking causes of cancer-related death by 2030 [1]. This highlights the limited efficacy of conventional anticancer therapies and makes it a priority to discover and propose new therapeutic strategies. Although the tumors can be surgically removed in only 10% of the cases, they most often relapse. For the inoperable tumors and the tumor relapses after surgery, chemotherapy alone or in combination with radiotherapy are the only alternatives. Currently, the main chemotherapies used, i.e. gemcitabine plus nab-paclitaxel, and the FOLFIRINOX regimen, have little effect with a median overall survival of less than 12 months in all studies [2–4]. Despite intense research, new therapies show limited efficacy in PDAC treatment [5].

In PDAC, the cancer cells are surrounded by a major stromal component consisting notably of a dense, organized extracellular matrix (ECM) [6]. In mice, disruption of the ECM through direct action on hyaluronan ECM components or by targeting stromal cells via the hedgehog pathway improves gemcitabine delivery and its effects on PDAC growth and mice survival [6, 7]. Several proteins and secreted factors regulate ECM organization. Lysyl oxidase (LOX) activity, exerted by the LOX, LOXL1, LOXL2, LOXL3, and LOXL4 proteins [8], is one of the most important regulator of the ECM. It is reported to increase migration, invasion, and metastasis dissemination through its capacity to regulate collagen cross-linking and ECM stiffening in different kinds of cancer [9–15]. This activity has also been implicated recently in senescence escape and cooperation with oncogenic signals to promote PDAC formation in mice [16].

Given the major ECM component in PDAC, the predominant role of LOX family activity in organizing the ECM, and the general pro-tumor functions of LOX family proteins, we have investigated its role in PDAC chemoresistance and its impact on gemcitabine distribution into the tumor.

## RESULTS

### Several LOX family members display increased expression in PDAC

**Figure 1:**
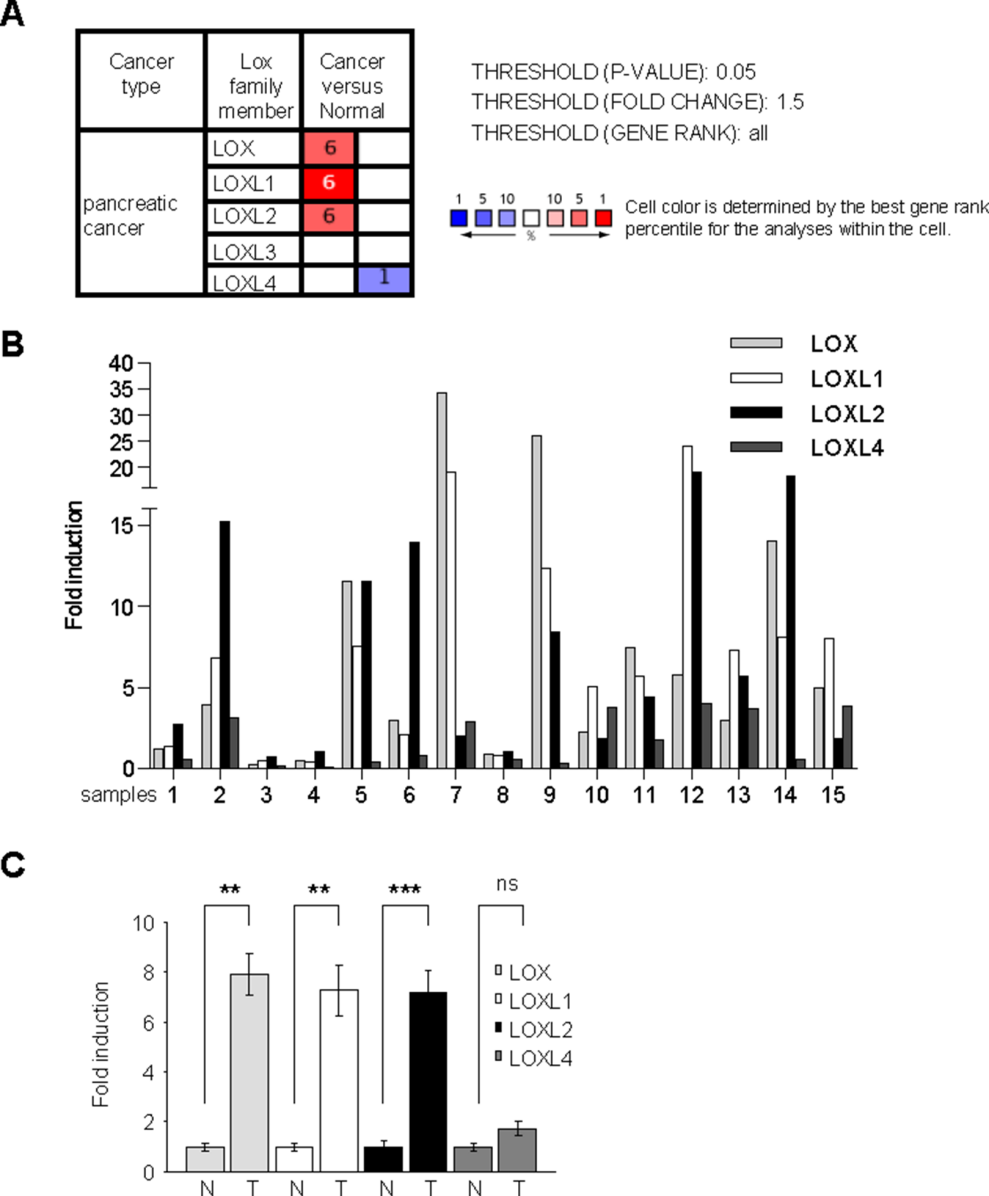
LOX activity increases in human PDAC (**A**) Summary of studies comparing levels of LOX, LOXL1, LOXL2, LOXL3, and LOXL4 transcripts in PDAC versus normal-tissue counterparts, obtained using the Oncomine database with the following settings: threshold *P*-value: 0.05; fold change: 1.5; gene rank all. The number indicated the number of study showing the same differential. Red color indicated an increase in the tumor whereas blue color indicated a decrease. (**B**–**C**) RNAs were extracted from both pancreatic normal and tumor tissues from the same patients and LOX, LOXL1, LOXL2, LOXL3, and LOXL4 transcript levels were assayed by qRT-PCR and normalized against HPRT1. LOXL3 was not detected. (B) Results are presented for each patient. (C) Results are presented as means ± SEM of LOX, LOXL1, LOXL2, and LOXL4 induction. *P*-values were determined with the Mann-Whitney *U* test.

To investigate transcript-level expression of the different LOX-family genes in PDAC, we first performed a search in the Oncomine database, a cancer microarray database used to compare gene expression levels in cancer versus normal tissues. This search revealed that LOX, LOXL1, and LOXL2 transcripts are upregulated in human PDAC (Figure 1A). These microarray data were confirmed by RT-qPCR performed on human PDAC samples and normal counterparts (described in [17]). On the one hand, the level of transcripts corresponding to each LOX-family member was normalized with respect to the HPRT1 transcript level and the fold induction calculated with respect to the level in the surrounding normal tissue (Figure 1B). On the other hand, these data were pooled and the mean fold induction for each LOX-family member was determined with respect to the mean of the controls (Figure 1C). As in the microarray database, increased levels of LOX, LOXL1, and LOXL2 transcripts were found in PDAC (Figure 1B, 1C). Together, these results demonstrate that LOX, LOXL1, and LOXL2 are upregulated in human PDAC.

### Increased LOX family activity promotes gemcitabine resistance

We next investigated whether increased synthesis and activity of LOX-family members in human PDAC might be involved in the well-known chemoresistance of PDAC. We first compared LOX family member expression in Colo-357 and MIA Paca-2 PDAC-derived cell lines. As for the tumors, the profile of expression of LOX family members was largely different between the two cell lines but expression was globally higher in MIA Paca-2 mainly for LOXL2 (Figure S1A). Accordingly, extracellular LOX family activity was also found higher in MIA Paca-2 when compared to Colo-357 (Figure S1B). Based on these results, we then choose to investigate whether an increased LOX family activity might render the Colo-357 more resistant to gemcitabine treatment. Colo-357 tumor growth was strongly inhibited by 80 mg/kg of gemcitabine whereas it was not significantly impacted by the 20 mg/kg of gemcitabine (Figure S2A). We then choose an intermediate dose of gemcitabine (40 mg/kg) to examine whether LOX family activity can modify tumor growth. To model increased LOX family activity in the tumors, and as LOX family members are secreted and can be produced by the cancer cells as well as by the stromal cells, we choose to add exogenously LOXL2 protein. In these settings, LOXL2 injection was active as fibrillar collagen increased (Figure S2B). Increased LOX family activity resulted in increased subcutaneous tumor growth in presence of gemcitabine (Figure S2C).

**Figure 2:**
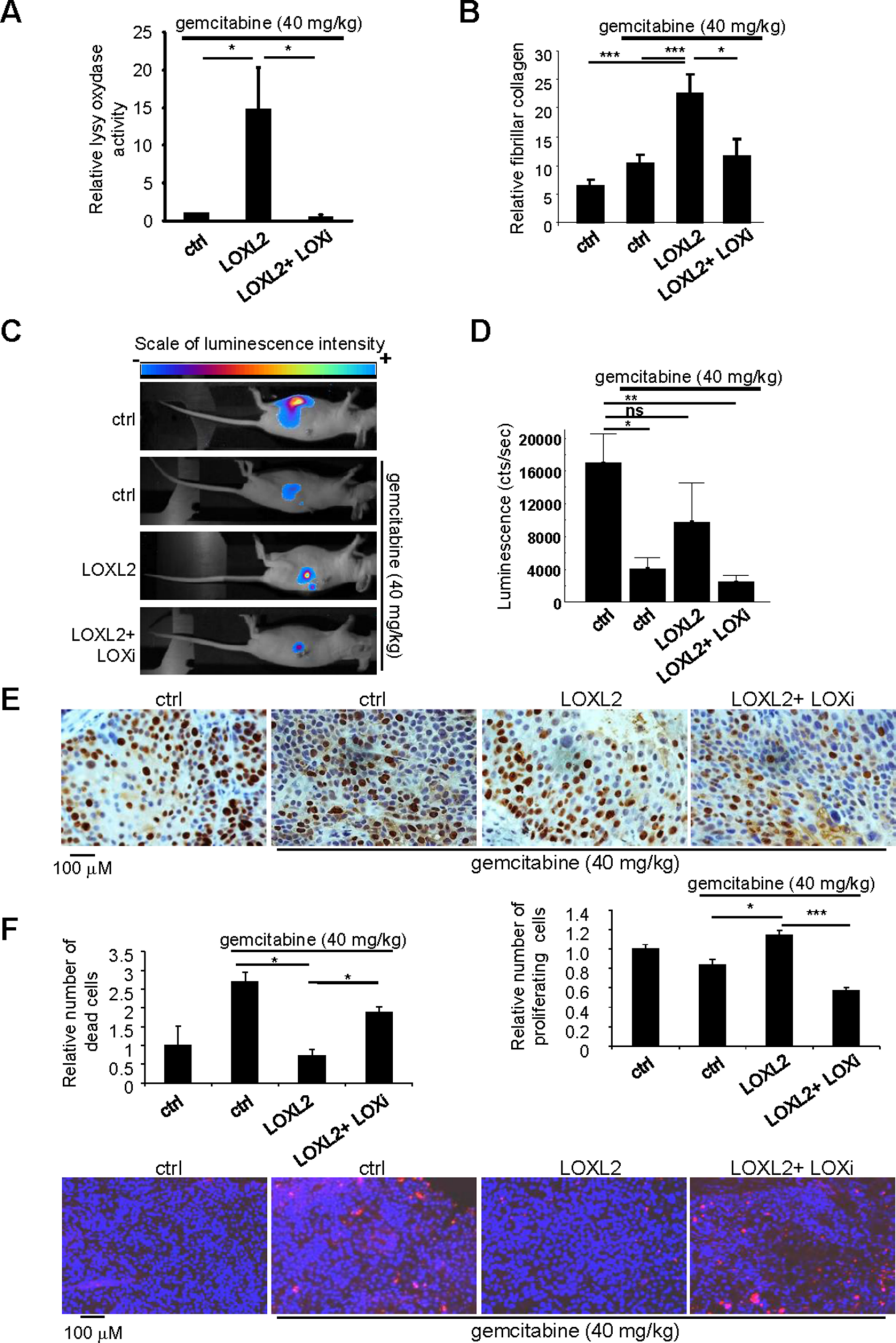
Modulating LOX family activity impacts the response to gemcitabine treatment One million Colo-357/Luc pancreatic cancer cells were orthotopically grafted into 6-week-old nude mice. The mice were left untreated or received IV injections of 40 mg/kg gemcitabine, alone or with LOXL2 or LOXL2 + LOXi injected IP when the tumors reached a mean luminescence of 800cts/s (*n* = 7 per group). Sixty-three days after the graft, (**A**) part of the tumors was used to measure LOX family activity or (**B**) the tumors were fixed, embedded, and tissue sections were stained with picrosirius red. Fibrillar collagen was analyzed by microscopy under polarized light. Values are means ± SEM. (**C**–**D**) Sixty-three days after grafting, tumor luminescence was measured. (C) Representative images of tumor development after 63 days are displayed for each condition; (D) luminescence is expressed as an integration of the average brightness/pixel unit (cts/s). (**E**) Immunohistochemical staining for the proliferation marker Ki-67 was performed. Representative photographs for each experimental condition are displayed. Ki-67-positive cells were counted in at least 25 fields. (**F**) A TUNEL cell death assay was performed. Representative photographs for each experimental condition are displayed. TUNEL-positive cells were counted in at least 15 fields. (A–E) Values are means ± SEM and the statistical test used was Mann-Whitney *U*.

We next performed experiments in which PDAC-derived cells expressing the luciferase gene were orthotopically injected in order to monitor tumor growth in the pancreas. When the tumors reached a mean luminescence of 800 cts/s, gemcitabine at 40 mg/kg was injected alone, with the LOXL2 protein, or with both the LOXL2 protein and the LOX family activity inhibitor (LOXi), to make sure the effect of the LOXL2 protein administered alone was due to its LOX activity. As expected, intraperitoneal LOXL2 injection lead to both, increased LOX family activity and fibrillar collagen, in tumors and, in the same time, co-injection of the LOXi completely prevented these effects (Figure 2A, 2B). In keeping with our results obtained with subcutaneous xenografts (Figure S2), LOXL2 injection prevented the tumor growth slowdown induced by gemcitabine, and LOXi countered this LOXL2-induced resistance to gemcitabine (Figure 2C, 2D).

Gemcitabine is a nucleoside analog that exerts its antitumor effects by inducing cell death and/or stopping cell proliferation. We therefore examined whether LOX family activity might regulate one or both of these gemcitabine-induced mechanisms. Immunohistochemical staining for the Ki67 proliferation marker demonstrated the ability of LOXL2 to prevent the proliferation-inhibiting effect of chemotherapy. Furthermore, this effect of LOXL2 was countered by LOXi treatment (Figure 2E). We then performed a cell death analysis, using the TUNEL assay. LOXL2 was found to prevent gemcitabine-induced cell death when used alone, but not in the presence of LOXi (Figure 2F).

Together these results indicate that LOX family activity promotes gemcitabine resistance by decreasing cell-death-promoting and proliferation-inhibiting effects of the drug.

### LOX activity decreases gemcitabine intra-tumoral distribution

**Figure 3:**
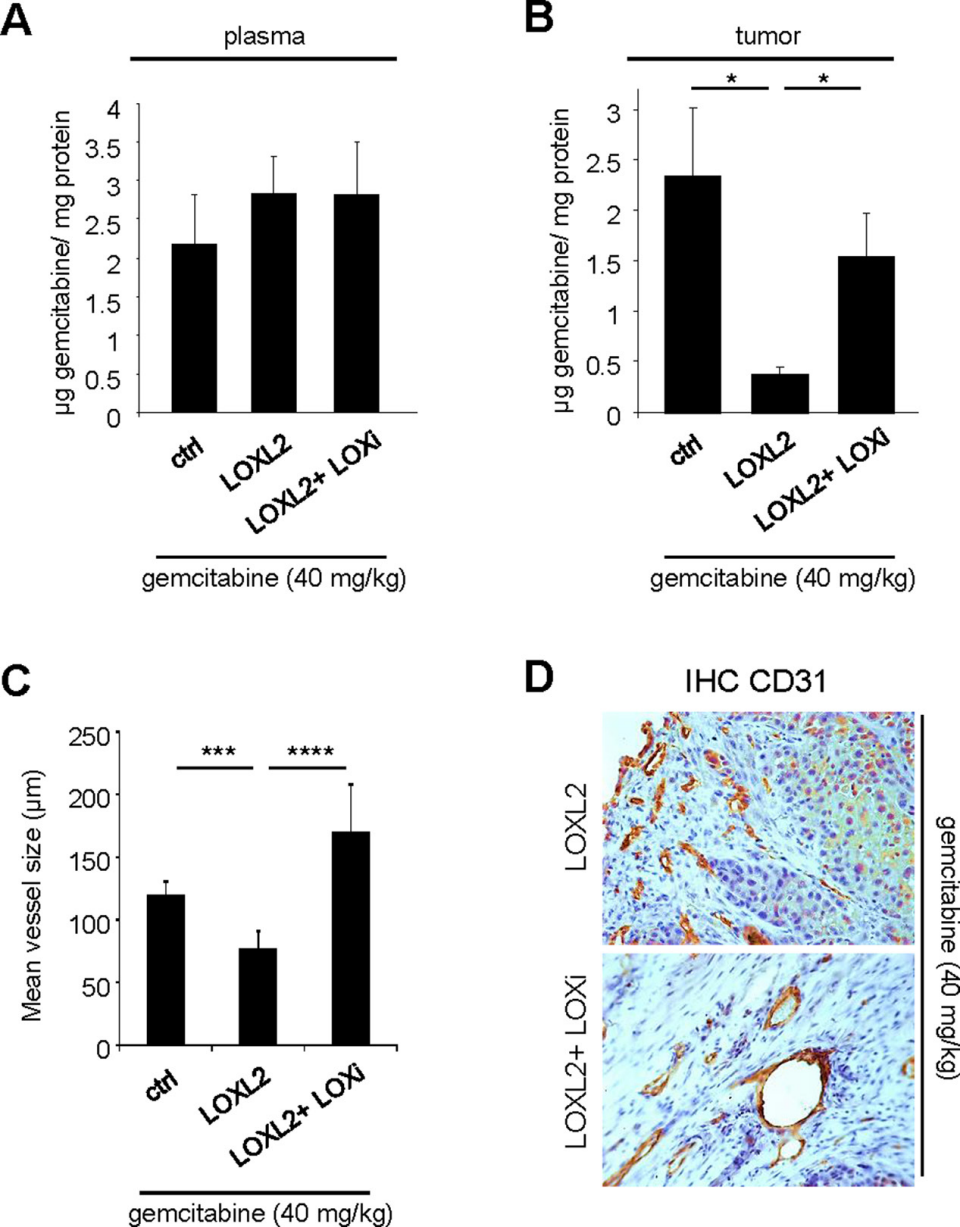
LOX family activity decreases gemcitabine distribution and favors vessels collapse (**A**–**B**) One hour before the endpoint of the orthotopic experiment described in Figure 3, 40 mg/kg gemcitabine was injected. At euthanasia, plasma and tumors of from the groups having received gemcitabine alone or in combination with LOXL2 or LOXL2+LOXi were removed for measurement of the gemcitabine concentration. The gemcitabine concentrations were determined (A) in the plasma and (B) in the tumor for each group and the results are expressed in μg of gemcitabine per mg protein ± SEM. The statistical test used was Mann-Whitney *U*. (**C**–**D**) Orthotopic tumors as described in Figure 3 were analyzed by immunohistochemical staining for CD31, an endothelial cell marker. (C) Vessel size was calculated with ImageJ software and (D) representative photographs are displayed. Values are means ± SEM and the statistical test used was Mann-Whitney *U*.

LOX family activity and collagen organization might create a biophysical barrier to gemcitabine distribution around and inside the tumor, as observed with other regulators of the ECM and tumor stroma [6, 7]. To test this possibility, we measured the gemcitabine concentration at the end of the orthotopic experiment. One hour before euthanasia, the mice were injected with gemcitabine at 40 mg/kg and the plasma and intratumoral gemcitabine levels were determined. As expected, similar levels of gemcitabine were found in the plasma under all conditions (Figure 3A). Interestingly, LOXL2-treated mice showed significantly lower intra-tumoral concentrations than the controls (Figure 3B). This effect of LOXL2 was counteracted by co-treatment with LOXi (Figure 3B). Together, these data demonstrate that resistance to gemcitabine treatment involves reduced diffusion of gemcitabine into the tumor.

ECM disruption by enzymatic ablation of hyaluronic acid ECM components or by hedgehog pathway inhibition improves gemcitabine delivery at least in part through an improved microvasculature [6, 7]. To investigate the role of LOX family activity in regulating the microvasculature, endothelial cells were stained with an anti-CD31 antibody to reveal the blood vessels. Interestingly, injections of the LOXL2 protein resulted in a strong decrease in blood vessel size (Figure 3C). This effect of LOXL2 appeared to depend on its activity, since the blood vessels appeared less collapsed in mice treated simultaneously with both LOXL2 and LOXi (Figure 3C, 3D).

Together, these results demonstrate that LOX family activity inhibits the intra-tumoral distribution of gemcitabine and inducing blood vessel collapse.

### Collagen organization is predictive of adjuvant gemcitabine benefit

To determine whether fibrillar collagen, a direct readout of LOX family activity [8, 18–20], might predict clinical outcomes, we analyzed these levels in a large series of patients who underwent curative intent resection for PDAC [21, 22]. The level of fibrillar collagen was determined by calculating the ratio between organization/quantity of collagen fibers in 86% of the tumors (*n* = 309). The clinical and pathological characteristics of the 309 patients are detailed in Table S1. In the whole population (*n* = 309), as in the subgroup of patients who did not receive an adjuvant treatment (*n* = 91), the level of fibrillar collagen fibers had no prognostic value in univariate and multivariate analysis (data not shown). In the whole population, there was a significant interaction between adjuvant Gemcitabine-based chemotherapy and the level of fibrillar collagen fibers for DFS [multivariate HR = 1.32; 95% CI = 1.08–1.59; *p* = 0.041] and OS [multivariate HR = 1.47; 95% CI = 1.09–2.08; *p* = 0.026].

**Figure 4:**
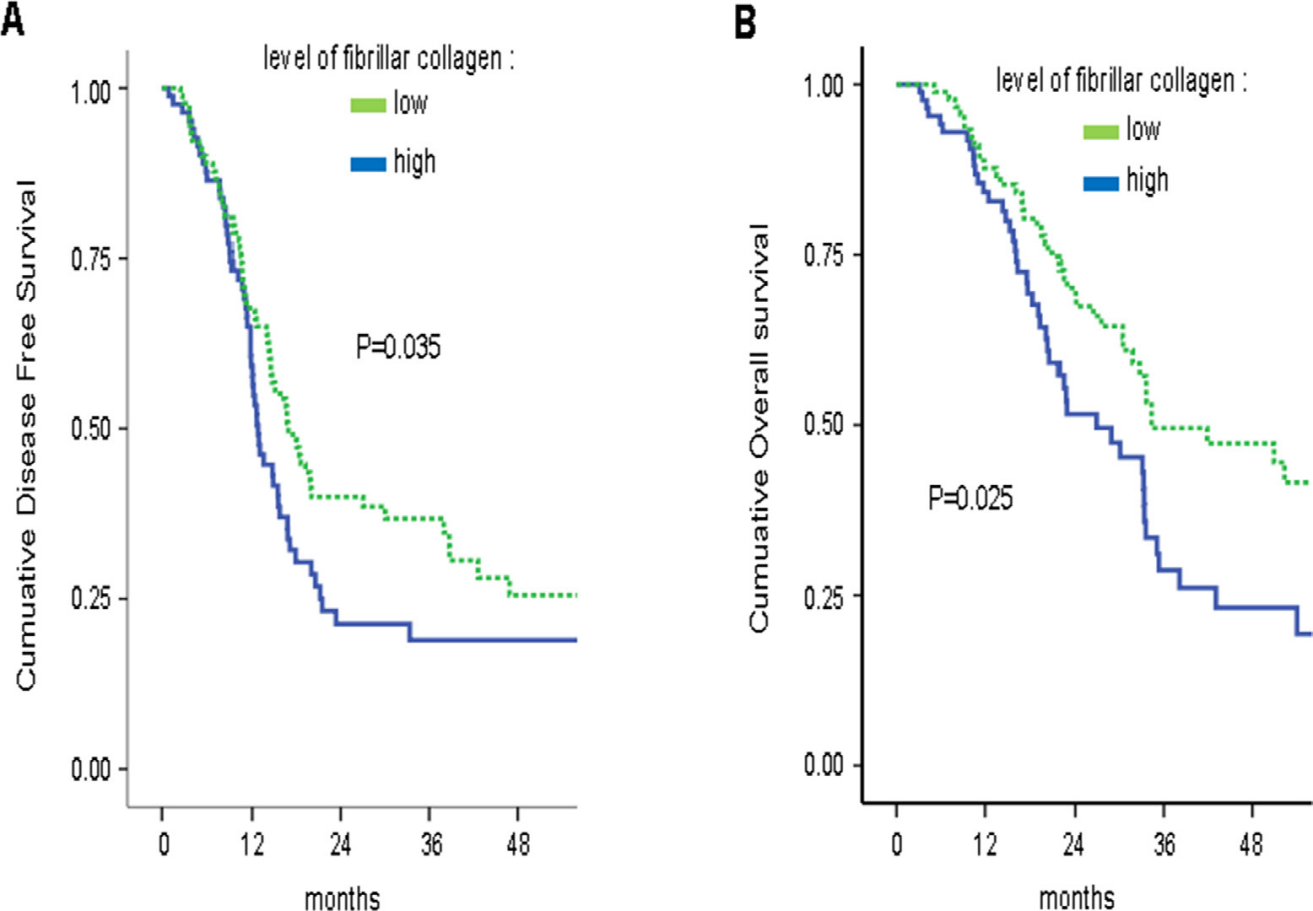
High fibrillar collagen level group displays lower survival during adjuvant gemcitabine-based therapy Level of fibrillar collagen was determined by calculating the ratio between fibrillar collagen and the quantity of collagen. Patients having received an adjuvant Gemcitabine-based chemotherapy were analyzed and samples were split in 2 groups, half of having the highest ratio and half having the lowest ratio. Kaplan Meier analyses were performed and represented (**A**) the disease free-survival and (**B**) the overall survival. *P* values were calculated using a log rank test.

**Figure 5:**
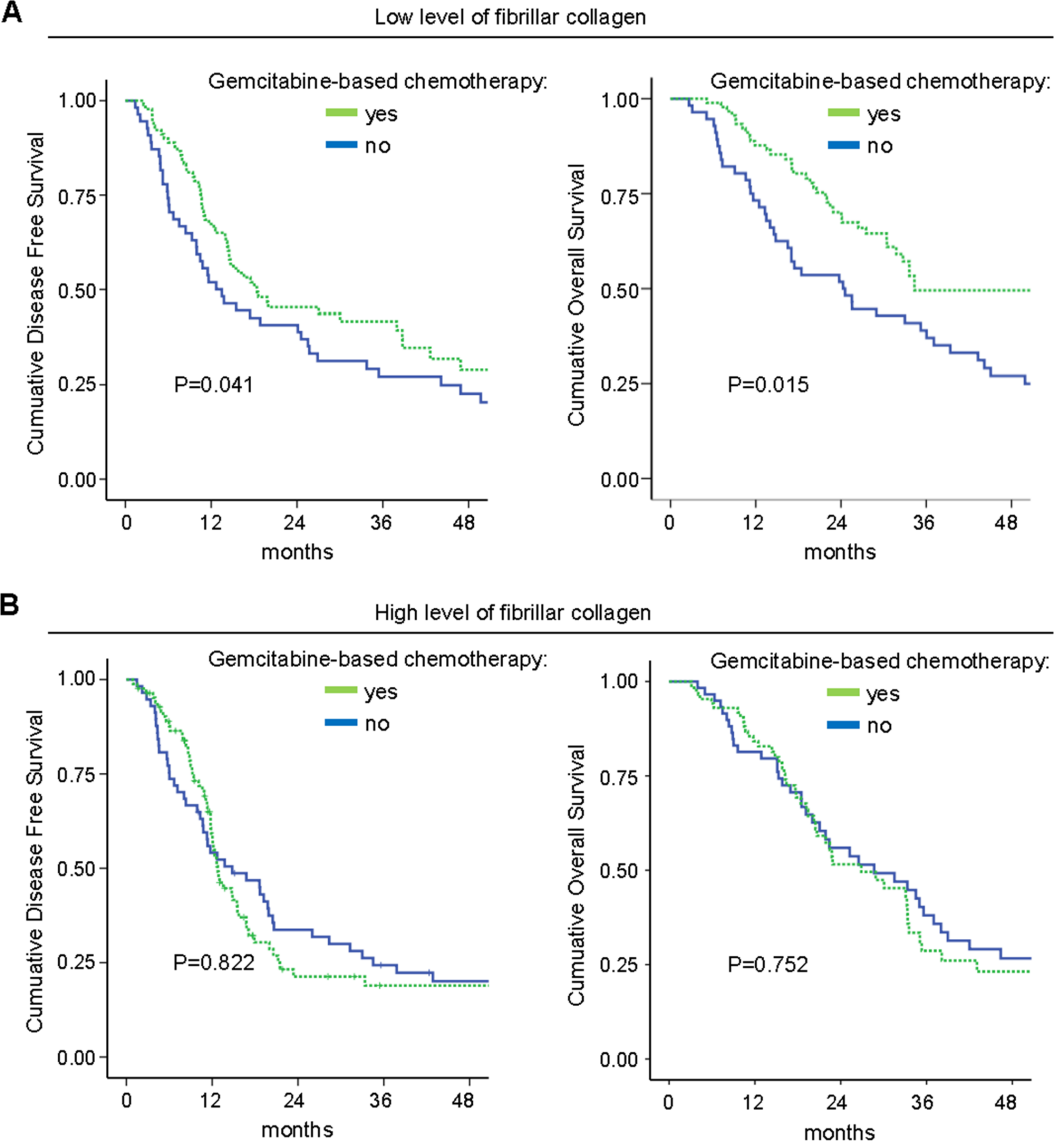
Fibrillar collagen level is a predictive factor for gemcitabine benefit Level of fibrillar collagen was determined by calculating the ratio between fibrillar collagen and the quantity of collagen. (**A**) Patients having a poor level of fibrillar collagen (mean 50% of the patients with the lowest collagen organization/quantity ratio) were analyzed and samples were split in 2 groups, having received or not an adjuvant gemcitabine-based chemotherapy. Kaplan Meier analyses were performed and represented the disease free-survival (left panel) and the overall survival (right panel). *P* values were calculated using a log rank test. (**B**) A similar analysis was performed in patients having a high level of fibrillar collagen (mean 50% of the patients with the lowest collagen organization/quantity ratio).

In the subgroup of patients who received an adjuvant “Gemcitabine-based chemotherapy” (*n* = 181), a high level of fibrillar collagen fibers was significantly correlated with a shorter DFS and OS in univariate analysis (Table S2, Figure 4). In multivariate analysis, a high level of fibrillar collagen fibers was an independent prognostic biomarker for DFS and OS (Table 1). In the univariate and multivariate analysis, no benefit in DFS or OS from adjuvant “Gemcitabine-based chemotherapy” was observed for patients with high fibrillar collagen level [DFS: multivariate HR = 1.15, 95% CI = 0.76–1.75, *p* = 0.504; OS: multivariate HR = 1.13, 95% CI = 0.72–1.79, *p* = 0.580], whereas adjuvant gemcitabine was of benefit in patients with a low fibrillar collagen level [DFS: multivariate HR = 0.63, 95% CI = 0.38–0.91, *p* = 0.014; OS: multivariate HR = 0.66, 95% CI = 0.42–0.95, *p* = 0.038] (Figure 5). Thus, a high level of fibrillar collagen fibers was a predictive biomarker of the absence of adjuvant gemcitabine benefit.

**Table 1:** Multivariate analysis in the subgroup of patients who received an adjuvant “Gemcitabine-based chemotherapy”

Variable	OS				DFS			
	HR*	95% CI	*p*-value	Bootstrap *x*1000	HR	95% CI	*p*-value	Bootstrap *x*1000
Margin positive	2.1	1.35–3.06	0.001	0.002	2.01	1.32–3.06	0.001	0.002
Node positive	-	-	-	-	1.20	0.72–2.00	0.483	0.447
Tumor differentiation	-	-	-	-	1.19	0.81–1.74	0.368	0.400
Fibrillar collagen High (> P50)	1.61	1.05–2.44	0.028	0.033	1.69	1.05–2.70	0.030	0.042

* HR of 1 indicates no difference between the two groups of patients, whereas an HR > 1 indicates an increased risk of death/failure

## DISCUSSION

LOX family activity has been implicated in tumor initiation and progression [9–14, 16, 23], but its role in tumor resistance, particularly in PDAC, to chemotherapy is not known. Here we demonstrate that increased levels of LOX-family members LOX, LOXL1 and LOXL2 expression are frequent in human PDAC samples and their activity are predictive of gemcitabine resistance *in vitro* and *in vivo* through modification of collagen organization, collapses of blood vessels and a decreased gemcitabine intra-tumoral distribution. Moreover, the level of fibrillar collagen appears as an independent predictive biomarker of adjuvant Gemcitabine based chemotherapy benefit.

We have previously demonstrated the involvement of LOX activity in escape from senescence and in PDAC formation [16]. Our present results further demonstrate its involvement in PDAC chemoresistance. We show that increased LOXL2 activity favors resistance to gemcitabine, whereas inhibition of its LOX activity promotes these antitumor effects. We further demonstrate that LOX family activity affects the efficacy of chemotherapy by regulating its diffusion into the tumor, as demonstrated by direct measurement of intra-tumoral gemcitabine concentrations. This conclusion is further supported by the observation that LOX family activity directly modulates all the responses induced by gemcitabine, such as cell death and proliferation, and that its inhibition does not increase the effect of very high doses of treatment (80 mg/kg).

The ability of LOX family activity to decrease the chemotherapeutic intra-tumoral concentration might be due to its ability to increase fibrillar collagen and then ECM stiffness, thus creating a physical barrier to gemcitabine diffusion. It might also be due to the vessel collapse observed in LOXL2-treated tumors, limiting gemcitabine arrival at the tumor site. LOX family activity has generally been associated with increased tumor angiogenesis [24–27]. In line with these results, we observed a slight increase in vessel number in LOXL2-treated tumors, disappearing in the presence of the LOX family activity inhibitor (Figure S3). Our results suggest that, although LOX famly activity might slightly increase vessel number, it strongly decreases vessel size. These results might be due to the fact that we examined the vessels after gemcitabine treatment, which might strongly affect tumor angiogenesis and its response to LOX family activity.

LOX family members, in particular LOXL2, can exert effects that do not depend on their enzymatic activity [28–30]. The fact that LOX family activity inhibitor prevents the effects of LOXL2 on collagen organization, gemcitabine resistance, vessel size, and gemcitabine intra-tumoral distribution strongly supports the view that LOXL2 exerts these effects via its enzymatic activity to promote PDAC chemoresistance.

As a result of their properties, LOX family members, in particular LOX and LOXL2, are viewed as promising targets of antitumor therapy. A LOXL2 mAb (simtuzumab) has been developed by Gilead [13] and used to treat PDAC in phase II trials [31]. Gilead has recently announced that this Ab, in combination with gemcitabine, failed to increase progression-free survival as compared to gemcitabine alone. This raises doubts as to the clinical utility of targeting LOXL2 to potentiate gemcitabine in PDAC. On the basis of our results showing that LOX, LOXL1, and LOXL2 expression can increase in PDAC and given the fact that the corresponding proteins share common substrates such as collagen and display similar other pro-tumoral effects [15], it is our opinion that targeting only one member of this family is likely to have no clinical benefit. Nevertheless, a recent study shows that specifically inhibiting LOX protein using a specific Ab improves PDAC chemosensitivity using a genetically engineered mouse model for PDAC [32]. Still designing a pan-LOX family members’ inhibitor might hold more promise than looking for specific inhibitors against each LOX family members. Used of LOX family inhibitors in combination with gemcitabine might also benefit of a biomarker, such as high level of fibrillar collagen, to choose the patient that might benefit to this therapy.

Hyaluronan destruction by hyaluronidase, another component of the ECM, has been showed to increase gemcitabine intra-tumoral distribution, through increased vasculature, and thus to potentiate gemcitabine efficiency in experimental models of PDAC [6, 33]. Hyaluronidase in combination with gemcitabine has recently been reported to give some benefit in phase II clinical trial but only in the patients displaying high levels of hyaluronan in the tumor [34], pointed out the importance to choose only the patients displaying the targeted alteration to get a clinical benefit. Taking into account the parallel effects of hyaluronidase and LOX family activity inhibition in increasing gemcitabine intra-tumoral distribution, it may be proposed that the combination of both, instead of the use of hyaluronidase or LOX activity inhibitor alone, will allow higher gemcitabine distribution and better clinical benefit.

In conclusion, our data demonstrate that LOX family activity can participate in the chemoresistance of PDAC by limiting the intra-tumoral distribution of a chemotherapeutic agent and the readout of its activity might be used as a biomarker to predict gemcitabine adjuvant benefit. Beside their implications for PDAC treatment, these results may lead to new clinical strategies for effective targeting of chemotherapeutic drugs to other cancer types.

## MATERIALS AND METHODS

### Cell culture

Human metastatic PDAC-derived cells (Colo-357) (ATCC) and virus-producing GP293 cells (Clontech) were cultured in DMEM (Life Technologies) containing Glutamax and supplemented with 10% FBS (Lonza) and 1% penicillin/streptomycin (Invitrogen). Upon receipt, the cells were thawed, amplified, and aliquots frozen. Experiments were performed on these aliquots within 4 months, without further authentication of the cell lines.

### RT-qPCR

Cells were lysed in TriReagent (Sigma) and total RNA was isolated via an acid-phenol extraction procedure using Phase Lock gel tubes (Eppendorf). RNA (2 μg) was reverse-transcribed with the First-Strand cDNA Synthesis Kit (GE Healthcare) according to the manufacturer's recommendations. Q-PCR experiments were carried out in a Light Cycler 2.0, in Light Cycler Taqman Master Mix (Roche) and with the Universal Probe Library (Roche Diagnostics). The following primers were used: LOX 5′-ggatacggcactggctactt-3′ and 5′-gacgcctggatgtagtaggg-3′, LOXL1 5′-gccagtggatcgacataacc-3′ and 5′-ccaaaacaat atactttgggttca-3′, LOXL2 5′-tgacctgctgaacctcaatg-3′ and 5′-tggcacactcgtaattcttctg-3′, LOXL4 5′-ggatacggcactg gctactt-3′ and 5′-ttgttcctgagacgctgttc-3′. The HPRT1 gene was used as a normalizer, with the 5′-tgaccttg atttattttgcatacc-3′ and 5′-cgagcaagacgttcagtcct-3′ primers.

### TUNEL assays

Tumor sections were treated with 4% PFA and 0.2% PBS-Triton and then incubated with TdT enzyme and biotinylated dUTP (Roche) in reaction buffer (200 mM potassium cacodylate, 25 mM Tris-HCL, 1 mM cobalt chloride). Biotinylated DNA was detected with Cy-3-coupled streptavidin (Interchim). The nuclei were stained with Hoechst dye (Sigma). The samples were analyzed by fluorescence microscopy and the number of TUNEL-positive cells was estimated with ImageJ software.

### Proliferation and vessel analysis

Fresh tumors were fixed for 48 hrs in 10% neutral buffered formalin (Sigma) and embedded in paraffin. For immunohistochemical staining, the paraffin-embedded tumors were serially sectioned at 4-μm thickness. After deparaffinization and rehydration in xylene and alcohol solutions, the slides were incubated in 5% hydrogen peroxide in sterile water to block endogenous peroxidases. The tissue sections were boiled in 10 mmol/l citrate buffer pH 6 (H3310, Vector laboratories) in a microwave oven for 20 min for heat-induced antigen retrieval. The slides were then incubated at room temperature for 1 hr with the primary anti-Ki-67 antibody (clone Tec-3, M7249, DAKO or CD-31, ab28364, Abcam) diluted in low-background solution (DAKO Real). After rinsing in PBS, the slides were incubated for 1 hr at room temperature with a biotinylated secondary antibody bound to a streptavidin peroxidase conjugate (Dako E0468). Bound antibody was revealed with a peroxidase substrate kit (Vectors Laboratories) and sections were finally counterstained with hematoxylin (Sigma).

### Generation of Luc-expressing PDAC cells

Colo-357/Luciferase cells were produced by infection with the MSCV Luciferase PGK-Hygro (Addgene plasmid 18782) retroviral vector. Production of retrovirus and infection of target cells were performed as described in [35]. Colo-357/Luciferase cells were hygromycin selected after infection.

### *In vivo* experiments

For subcutaneous xenografts, 5 × 10^6^ Colo-357 cells per mouse were injected into the right flanks of 6-week-old female NMRI nude mice (Janvier) in 200 μl PBS containing 25% Matrigel (BD Biosciences). Tumor size was measured with an electronic caliper and tumor volume was calculated with the formula (length × width × depth)/2.

For orthotopic xenografting into the pancreas, NMRI nude mice were injected under general anesthesia with isoflurane. One million Colo-357/Luciferase cells were suspended in 30 μl PBS containing 5% Matrigel and injected with a 31-gauge needle. After surgery, the peritoneum was sutured with an absorbable silk suture and the skin with Michel suture clips. During the first 24 hrs, the animals were maintained under analgesia. The mice were monitored for body weight and tumor development with the NightOWL *in vivo* imaging system (Berthold). Briefly, each mouse received a 2 mg dose of luciferin (Promega) in sodium chloride solution by intraperitoneal injection. Ten minutes later, luminescence was quantified in the anesthetized mouse for ten minutes to reach the maximum value.

In all *in vivo* experiments, gemcitabine (Eli Lilly) was administered by intravenous injection into the tail vein at the dosages indicated in the figure legends. Ten nanograms of recombinant LOXL2 protein (R & D systems) was injected directly into the tumor, in the case of subcutaneous xenografts, or intraperitoneally, in the case of pancreatic xenografts. The LOX familty activity inhibitor beta-aminopropionitrile (BAPN) (Sigma) [36–38] was administered intraperitoneally each day (at 100 mg/kg in 100 μl 0.9% sodium chloride), alone or in combination with recombinant LOXL2 protein.

The mice were housed and bred in the AniCan dedicated pathogen-free animal facility (CRCL, Lyon, France). All experiments were performed in accordance with the animal care guidelines of the European Union and with French legislation and were approved by the local Animal Ethics Evaluation Committee (CECCAPP).

### Quantification of gemcitabine in tumors and plasma

One hr before euthanasia, mice were injected in the tail vein with 100 μl gemcitabine solution at 40 mg/kg. Blood samples were centrifuged in the presence of heparin to separate the plasma and conserved in liquid nitrogen. Tumor samples were snap-frozen in liquid nitrogen to maintain the stability of gemcitabine before cryogenic grinding (Cryotec). Throughout the process, the samples were maintained at −80°C. Gemcitabine was then quantified in the plasma and tumor samples by hydrophilic interaction liquid chromatography hyphenated to tandem mass spectrometry, and the results were normalized with respect to the protein content determined by the Bradford method. In this LC-MS/MS, the chromatographic separation was carried out in the isocratic mode, with a Hilic-Atlantis^®^ analytical column (2.1 mm × 150 mm; 3 μm – Waters) and with [^13^C, ^15^N_2_]-gemcitabine used as internal standard. The eluent was a mixture of acetate buffer (pH 5, 100 mM) and acetonitrile (15/85 v/v). Mass spectra were acquired with a triple-quadrupole mass spectrometer (Quantum-Ultra; Thermo Electron, San Jose, USA). An electrospray ionisation source was used in the positive mode and gemcitabine and [^13^C, ^15^N_2_]-gemcitabine were quantified, respectively, by selected reaction monitoring of the 264→112 and 267→ 115 transitions. Ten microliters of plasma or 100 μL of tissue solution was introduced into a conical centrifuge tube. After protein precipitation with 300 μL cold acetonitrile, the sample was mixed with 50 μL internal standard solution and then centrifuged for 10 min at 13 000 g and 6°C. The clear supernatant was transferred to a glass vial. The concentration ranges for the calibration curves were 0.05 to 4 μg/ml for plasma and 0.1 to 4 μg/ml for tumor samples.

### Collagen fiber organization

Paraffin-treated sections were deparaffinized and stained for 1 hr in 0.1% picrosirius red (Direct Red 80, 365548, Sigma) in picric acid solution (P6744, Sigma). Counterstaining was performed with Weigert hemotoxylin (acid condition) (HT1079, Sigma). Collagen fiber deposits and fibrillar collagen were visualized, respectively, by normal light and polarized light microscopy, and quantified with ImageJ software. Fibrilar collagen level, a direct readout of LOX family activity [8, 18–20], was defined by the ratio fibrillar/quantity and calculated for statistical analysis: a low score indicates a poor level of collagen fiber organization whereas a high score indicates a high collagen fiber organization. The same methodology was used to analyze collagen fiber organization in orthotopic xenograft models and in TMAs from human PDAC.

### LOX family activity assays

LOX family activity assays were performed with the Amplex Red Monoamine Oxidase Assay Kit (molecular probes, Invitrogen). For the supernatant activity, each cell lines were plated at the same density (750.000 cells per 10 cm dishes) and 48 hours after, cell mediums were collected. Measured were performed on 20 μl of medium. For tumor samples, LOX family activities were measured on 2 μg of proteins after cryogenic grinding. Each sample was used and measured according to manufacturer's instructions and as previously described in [16]. Amplex red was excited at 530 nm and emission was measured at 590 nm using Tecan M1000 PRO. Assays were run in sextuplicate and activity were reported as a mean ± S.E.M of all assays.

### Statistical analysis

Values are presented as means ± S.E.M unless stated otherwise. Data obtained from independent groups were compared by the nonparametric Kruskal-Wallis (more than two groups) or Mann-Whitney *U* test (two groups) (*P < 0.05, **P < 0.01, ***P < 0.001). Statistical analyses were performed with Statistica software (Statsoft). Statistics for the TMA were explained below in the TMA paragraph.

### Tissue microarray (TMA)

Prognostic and predictive values of collagen fiber organization have been assessed in a large series of patients who underwent curative intent resection for PDAC in four university centers specialized in PDAC treatment. Patient selection, clinicopathologic data records, characteristics of the population and modalities of TMA construction have been previously described [21, 22, 39, 40]. Because of TMA depletion, a total of 359 patients among the 471 original patients of the whole series were included in this study. Appropriate ethic committees approved the study.

Overall Survival (OS) was defined as time from surgery to death resulting of any cause. Disease Free Survival (DFS) was measured from the date of surgery to the date of relapse or death. Survival curves were estimated using the Kaplan-Meier technique and compared using the log-rank test. The Cox proportional hazard regression model was used for both univariate and multivariate analyses and for estimating the hazard ratio with 95% confidence interval. For multivariate analysis, internal validation was checked using bootstraping (1000 replications).

For prognostic and predictive analyses of level of fibrillar collagen, two groups of patients were defined according to the median value of the organization/quantity ratio: high level of fibrillar collagen (patients with collagen organization/quantity ratio > median value) and low level of fibrillar collagen (patients with collagen organization/quantity ≤ median value).

Prognostic value of the fibrillar collagen level was first evaluated in the whole population then in the subgroup of patients who did not receive any adjuvant treatment.

In the whole population, an interaction test between the fibrillar collagen level and the administration of adjuvant “Gemcitabine-based chemotherapy” was performed using the Cox model to calculate the HRs (Hazard Ratio) with 95% CIs. Subsequently, the predictive value of the fibrillar collagen was evaluated in the subgroup of patients who received a “Gemcitabine-based chemotherapy”. Analyze of the predictive value of fibrillar collagen was carried out according to two subgroups defined by the median ratio organization/quantity.

Based on univariate Cox significance level of 0.1, clinical variables and biomarker were incorporated into the Cox models for multivariate analysis. A backward selection was then applied to construct the final multivariate model (keeping all the variables with a *p* value ≤ 0.10).

For each test, statistical significance was set at a two-sided *p* value of < 0.05. Statistical analyses were done with Stata V11 software.

## SUPPLEMENTARY MATERIALS FIGURES AND TABLES


