# Supplementary material for: Lysyl oxidase family activity promotes resistance of pancreatic ductal adenocarcinoma to chemotherapy by limiting the intratumoral anticancer drug distribution
Source: Oncotarget. 2016 Apr 1;7(22):32100–12. doi: 10.18632/oncotarget.8527 (PMC5078000; doi:10.18632/oncotarget.8527)
Supplement: Supplementary file 1 [file oncotarget-07-32100-s001.pdf]

# Lysyl oxidase family activity promotes resistance of pancreatic ductal adenocarcinoma to chemotherapy by limiting the intratumoral anticancer drug distribution

## Supplementary Materials

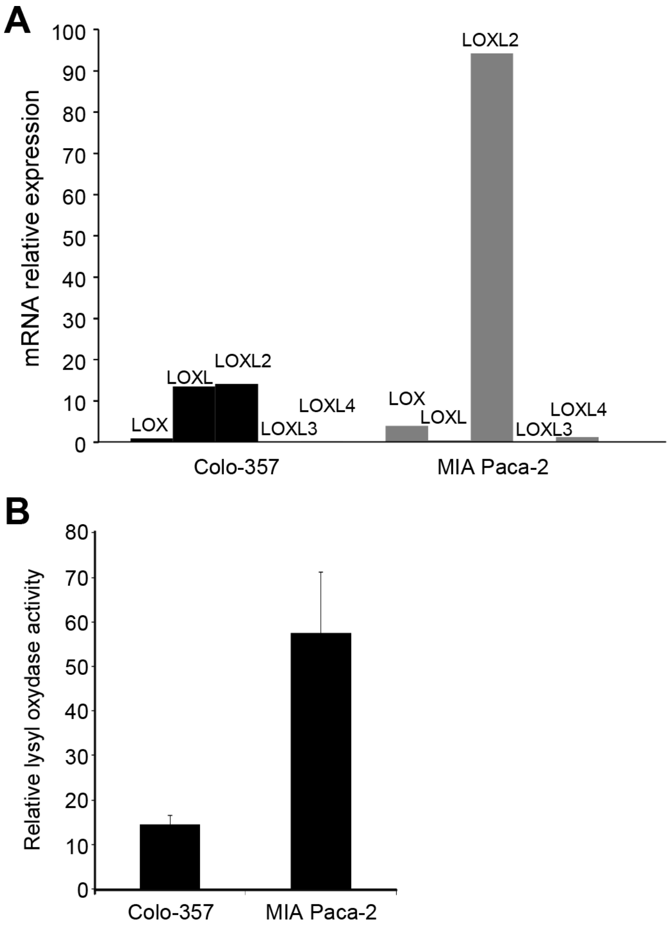


**Supplementary Figure S1: Expression and activity of LOX family members.** (**A**) RNAs were extracted from the indicated PDAC-derived cell lines and LOX, LOXL1, LOXL2, LOXL3, and LOXL4 transcript levels were assayed by qRT-PCR and normalized against HPRT1. (**B**) Supernatant of the indicated cells lines were removed and LOX family members activity measured.

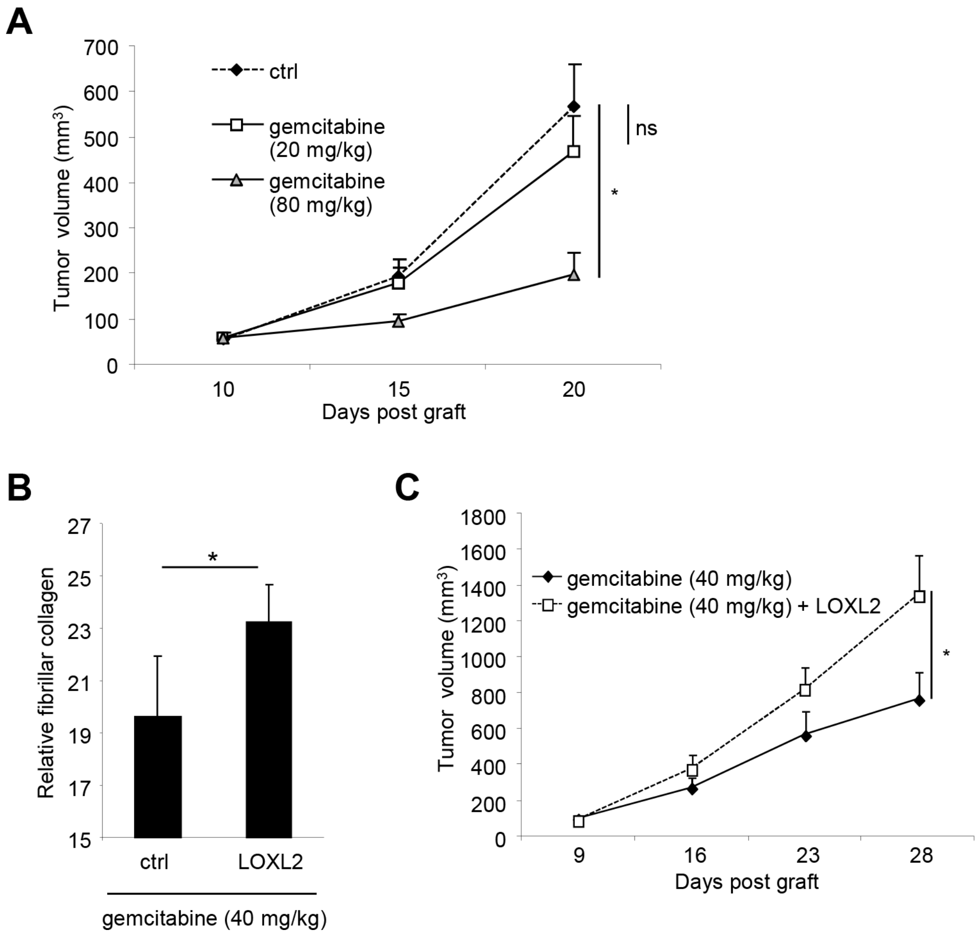


**Supplementary Figure S2: Response to gemcitabine treatment depends of lysyl oxidase level activity.** Five million Colo-357 pancreatic cancer cells per mouse were grafted into the right flanks of 6-week-old nude mice. The mice were subjected to the indicated treatment when the tumors reached a mean size of 50 $mm^3$ (**A**) or of 100 $mm^3$ (**B**–**C**). (A) Mice were intravenously injected every 3 days with the indicated dose of gemcitabine and tumor volumes were calculated ($n$ = 12 per group). Values are means ± SEM. (B–C) Mice were treated every two days by IV injection of 40 mg/kg gemcitabine, alone or in combination with 10 ng recombinant LOXL2 injected directly into the tumor ($n$ = 5 per group). (B) Tissues were picrosirius red stained and microscopically analyzed under polarized light to quantify cross-linked collagen. Relative levels of cross-linked collagen are presented as means ± SEM. (C) Tumor volumes were calculated and means ± SEM are presented. In each panel, values are means ± SEM and the statistical test used was Mann-Whitney $U$.

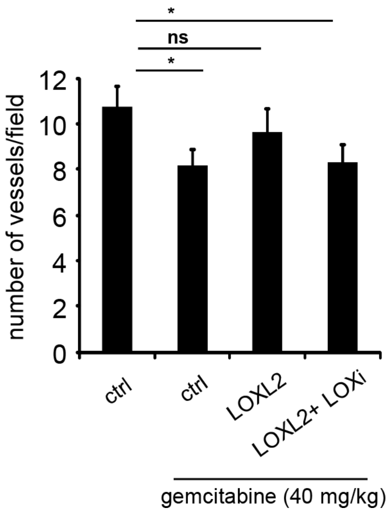


**Supplementary Figure S3: LOX family activity increases vessel number.** One million Colo-357/Luc pancreatic cancer cells per mouse were orthotopically grafted into 6-weekold nude mice. The mice were left untreated or received IV injections of 40 mg/kg gemcitabine. alone or with IP-injected LOXL2 or LOXL2 + LOXi. when the tumors reached a mean luminescence of 800 cts/s ($n$ = 7 per group). Sixty-three days after grafting. the tumors were processed for immunohistochemical CD31 staining. The mean number of vessels per field was calculated and statistical analysis was performed with the Mann-Whitney $U$ test.

**Supplementary Table S1: Patients' characteristics**

| Variable | No. of Patients | % |
|---|---|---|
| Gender | | |
| Female | 147 | 47.6 |
| Male | 162 | 52.4 |
| Age, years | | |
| Median | 62.3 | |
| range | 37–87 | |
| Mean | 63.2 | |
| SD | 10.1 | |
| Resection margins | | |
| R0 | 239 | 77.3 |
| R1 | 70 | 22.7 |
| Tumor Differentiation | | |
| Well | | |
| Moderate | 135 | 43.7 |
| Poor | 121 | 39.2 |
| Unknown | 40 | 12.9 |
| Tumor Differentiation | | |
| Well/moderate | 256 | 82.8 |
| Poor | 40 | 17.2 |
| Tumor stage | | |
| T1/T2 | 57 | 18.4 |
| T3 | 252 | 81.6 |
| Lymph Nodes | | |
| Negative, N0 | 78 | 25.2 |
| Positive, N1 | 231 | 74.8 |
| Tumor Stage | | |
| I | 7 | 2.3 |
| IB | 13 | 4.2 |
| IIA | 51 | 16.5 |
| IIB | 238 | 77.0 |
| Perineural invasion | | |
| Negative | 94 | 30.4 |
| Positive | 213 | 69.6 |
| Lymphovascular invasion | | |
| Negative | 125 | 40.5 |
| Positive | 184 | 59.5 |
| Adjuvant therapy | | |
| Yes | 212 | 70.6 |
| No | 91 | 29.4 |
| Type of adjuvant therapy | | |
| Gemcitabine-based chemotherapy | 181 | 85.4 |
| Other | 31 | 14.6 |

abbreviations: SD, standard deviation.

**Supplementary Table S2: Univariate analysis in the subgroup of patients who received an adjuvant "Gemcitabine-based chemotherapy"**

| | $n$ = 181 | | | $n$ = 174 | | |
|---|---|---|---|---|---|---|
| **Variable** | **No. events** | **Median OS (months)** | **Log-Rank *p*-value** | **No. events** | **Median DFS (months)** | **Log-Rank *p*-value** |
| Gender | | | | | | |
| Female | 45/89 | 33.1 | | 53/86 | 15.5 | |
| Male | 46/92 | 33.6 | 0.720 | 63/89 | 14.7 | 0.611 |
| Resection margins | | | | | | |
| R0 | 61/133 | 35.1 | | 79/130 | 16.8 | |
| R1 | 30/48 | 20.5 | < 0.001 | 37/45 | 11.3 | < 0.001 |
| Tumor Differentiation | | | | | | |
| Well | 36/78 | 35.1 | | 46/77 | 18.6 | |
| Moderate | 37/70 | 32.7 | | 47/68 | 14.3 | |
| Poor | 14/27 | 22.9 | 0.241 | 21/26 | 11.3 | 0.026 |
| Tumor Differentiation | | | | | | |
| Well/moderate | 73/148 | 33.6 | | 93/145 | 16.6 | |
| Poor | 14/27 | 22.9 | 0.182 | 21/26 | 11.3 | 0.020 |
| Tumor stage | | | | | | |
| T1/T2 | 16/25 | 33.6 | | 17/25 | 15.1 | |
| T3 | 75/156 | 33.3 | 0.642 | 99/150 | 12.6 | 0.820 |
| Lymph Nodes | | | | | | |
| Negative, N0 | 17/37 | 35.1 | | 19/35 | 20.0 | |
| Positive, N1 | 74/144 | 32.7 | 0.268 | 97/140 | 14.5 | 0.049 |
| Perineural invasion | | | | | | |
| Negative | 28/61 | 34.3 | | 35/60 | 18.4 | |
| Positive | 63/120 | 30.5 | 0.699 | 81/115 | 14.5 | 0.103 |
| Lymphovascular invasion | | | | | | |
| Negative | 28/61 | 34.3 | | 26/43 | 17.9 | |
| Positive | 63/120 | 31.8 | 0.160 | 89/130 | 14.1 | 0.138 |
| Level of fibrillary collagen P50 (median) | | | | | | |
| High (> P50) | 46/89 | 26.9 | | 56/84 | 12.8 | |
| Low (< P50) | 44/91 | 34.3 | 0.025 | 59/90 | 16.9 | 0.035 |
